# K2D2: Estimation of protein secondary structure from circular dichroism spectra

**DOI:** 10.1186/1472-6807-8-25

**Published:** 2008-05-13

**Authors:** Carolina Perez-Iratxeta, Miguel A Andrade-Navarro

**Affiliations:** 1 Ontario Genomics Innovation Centre, Ottawa Health Research Institute, 501 Smyth, Ottawa, ON, K1H 8L6, Canada; 2 Department of Cellular and Molecular Medicine, Faculty of Medicine, University of Ottawa, Canada; 3 Computational Biology and Data Mining Group, Max Delbrück Centre for Molecular Medicine, Berlin, Germany

## Abstract

**Background:**

Circular dichroism spectroscopy is a widely used technique to analyze the secondary structure of proteins in solution. Predictive methods use the circular dichroism spectra from proteins of known tertiary structure to assess the secondary structure contents of a protein with unknown structure given its circular dichroism spectrum.

**Results:**

We developed K2D2, a method with an associated web server to estimate protein secondary structure from circular dichroism spectra. The method uses a self-organized map of spectra from proteins with known structure to deduce a map of protein secondary structure that is used to do the predictions.

**Conclusion:**

The K2D2 server is publicly accessible at . It accepts as input a circular dichroism spectrum and outputs the estimated secondary structure content (alpha-helix and beta-strand) of the corresponding protein, as well as an estimated measure of error.

## Background

Circular dichroism (CD) spectroscopy is a widely used technique to analyze the secondary structure of proteins in solution. It is based on the dependence of the optical activity of the protein in the 170–240 nm wavelength with the backbone orientation of the peptide bonds with minor influences from the side chains [1]. Different types of secondary structure producing characteristic spectra, the spectrum of a given protein can be used to estimate its percentage content on the major secondary structure types. During the past 30 years, many methods that address this problem have been developed, which apply a variety of approaches from singular value decomposition to optimization algorithms, regression or neural networks [2-13]. One of these methods is K2D [8]. It uses a self-organizing map (SOM) algorithm, a type of neural network. Spectra from proteins with solved tertiary structure are used as training set to produce the SOM. From the resulting map of spectra, "secondary structure maps" are derived. The secondary structure map is directly related to the spectra SOM and this relation is applied to estimate the percentages of content in alpha helix and beta strand of a protein given its CD spectrum.

Here we present K2D2, a re-implementation of K2D using the latest version of the SOM_PAK package [14]. K2D2 accepts a broader wavelength range for the input spectra, 190 to 240 nm further to the 200 to 240 nm wavelength range originally accepted by K2D, and has been trained with a much extended set of spectra. As a result K2D2 displays a considerable advance in performance over K2D.

## Implementation

### CD spectra and structural data

A number of 43 CD spectra from proteins was obtained from CDPRO reference set CDDATA.43 constructed from different contributors (W. C. Johnson and [15-17]). It consists of spectra for soluble proteins with a variety of secondary structure composition: *mainly alpha *(myoglobin, hemoglobin, hemerythrin, etc.), *mainly beta *(elastase, tumor necrosis factor, alpha-chymotrypsin, etc.) and *alpha/beta *(triose phosphate isomerase, lactate dehydrogenase, lysozime, thermolysine, etc) (see Table 1). Our attempt to use a larger CD data set that includes 13 transmembrane proteins resulted in a poorer performance of the method, which suggests that these proteins require a specialized method trained only with transmembrane proteins. The difficulties in predicting the secondary structure of transmembrane proteins with CD methods trained with globular proteins have been noted before [18].

**Table 1:** Performance on benchmarks for K2D and K2D2.

				**α**		**β**	
**METHOD**	**Ref-set**	**Eval-set**	**WRange**	**RMSD**	**r**	**RMSD**	**r**

**K2D**	24	24	200–240	0.11	0.91	0.14	0.73
		43	200–240	0.12	0.91	0.13	0.66
**K2D2**	49	43	200–240	0.10	0.90	0.10	0.78
		43	190–240	0.08	0.93	0.09	0.82

**Ref-set**: number of proteins in the reference or training set. **Eval-set**: number of proteins used to evaluate the performance. **Wrange**: spectra wave length range. **α**: alpha helix. **β**: beta strand. **RMSD**: root mean square deviation. **r**: Pearson correlation coefficient. Results for K2D evaluated with 24 proteins are taken from [8].

We selected best resolution tertiary structures corresponding to the proteins in the reference set from the Protein Data Bank (PDB) [19]. We used the DSSP program [20] on the PDB files in order to assign secondary structure class to the individual amino acids in every protein in the reference set. We assigned alpha-helix to the protein residues labeled as H and beta-strand to those labeled E and then computed the fraction of amino acids in the protein in each conformation (see Table 1). In addition to the CDDATA.43 spectra, we included in the training set six additional reference spectra from [21]: three spectra of poly(L-lysine) in aqueous solution in alpha, beta and random conformations, and three model spectra in alpha, beta and random conformation constructed from 15 proteins [16].

### Spectra SOM and secondary structure maps

**Figure 1:**
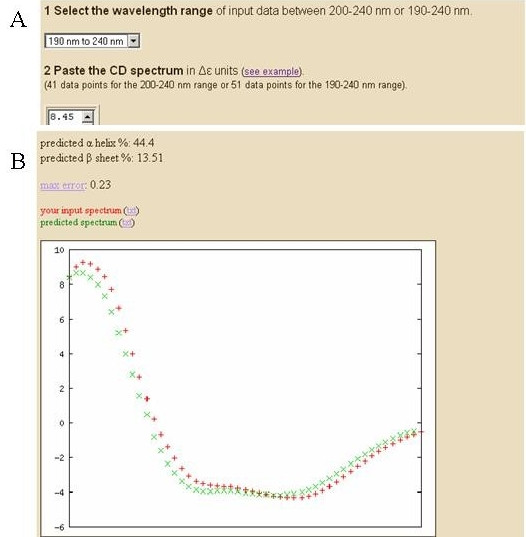
**K2D2 web server input and output.** (A) Window for input spectrum. (B) Comparison of input and predicted spectra.

A map of 18 × 18 neurons was trained with the 49 CD spectra using the SOM_PAK package [14]. Small variations on the map size and training parameters that produced smooth maps did not produce big differences in performance. The final map was produced by averaging 100 randomly started maps. Once the spectra SOM is obtained we produce two "secondary structure maps", one for alpha-helix and another for beta-strand. We start with grids of 18 × 18 nodes (same size as the SOM), and we compare each spectrum in the training set with the weight vectors associated to the neurons of the SOM. Given a spectrum, we find its "closer" neuron in the SOM map, and we assign the fraction of secondary structure of the corresponding protein to the equivalent (same coordinates) node in the grid. In order to produce smooth maps (see Figure 1), instead of considering only the closer neuron in the spectra SOM we take into account a number n of the closest neurons, and the final value of secondary structure fraction is the linear combination of the values of the respective neurons weighed by the inverse of their distances. The inclusion of more than 6 neighboring neurons produced the best results. Better performance was obtained if the extra six reference spectra from [21] were not included in the computation of the secondary structure map, although performance decreased if we removed them as well from the training set of the spectra SOM. Therefore, we kept them for the training.

### Estimated maximum error

In principle, the more similar a given spectrum is to its closest SOM spectra node, the better would be the prediction. In other words, if a spectrum is very different to anything the method has "previously seen" (as for training set), results cannot be expected to be very accurate. To provide users with an estimate of the maximum total error of the prediction (as sum for the errors for the alpha and beta predictions) we used the distances to the closest node map and the corresponding observed total errors from the benchmark. At a given distance, the maximum error is the largest total error that was observed in the benchmark. Thus, the total error for the prediction is expected to be less than the estimated maximum error. If the distance is larger than anything observed in the benchmark, a message is given indicating that an estimation of maximum error is not possible; in this situation the structure prediction should not be taken into account.

### Web server

K2D2 can be accessed at K2D2 site [22]. Users must choose the input wavelength range (200–240 nm or 190–240 nm) and provide the spectrum of the problem protein (see Figure 1A). Spectra must be in Δε units. As results are better for the 190–240 nm wavelength range, this option is recommended if the user can supply spectra in this range, although we maintain the short range input as it is sometimes difficult to obtain the former. The results consist of the estimated values for percentages of residues in alpha-helix and beta-strand, an estimated error for the prediction, and a graphic comparing the predicted spectrum with the user input (see Figure 1B). The plot provides a visual assessment of the accuracy of the prediction.

## Results and Discussion

The performance of K2D2 was measured in a left-one-out benchmark, comparing real and predicted values, by means of the Pearson correlation coefficient (r) and the root mean square deviation (RMSD). We obtained averages of r of 0.93 for alpha and 0.82 for beta, and average values of RMSD of 0.08 and 0.09, respectively (see Table 1). In comparison, K2D was reported to produce average r values of 0.91 for alpha and 0.73 for beta, and average RMSD values of 0.11 and 0.14, respectively [8]. The performance of K2D2 for alpha helix did not improve much, something to expect, as K2D's prediction was already very good. In contrast, the prediction for beta strand was much improved. Furthermore, K2D was originally tested with only 24 proteins, and when evaluating it with the expanded set of 43 proteins we observed an even bigger difference (see Table 1). Thus, K2D2 produces significantly better predictions than K2D.

## Conclusion

We have presented K2D2, a re-implementation of the K2D method for prediction of protein secondary structure from CD spectra. By using a larger wavelength range and larger dataset training, K2D2 represents an important improvement over K2D.

As for K2D [8], we chose to offer predictions for alpha and beta percentages and not for other structures that are sometimes considered by similar prediction algorithms. Beta-turns are predicted by various methods with mild success (see Table 2). Recent publications point out that protein CD spectra does not have good predictive value for beta-turns [23], the reasons being the small number of residues in turns and the heterogeneity of their conformation [24].

**Table 2:** Reported performance for different implementations of published methods.

				**α**		**β**		**T**		**U**	
**METHOD**	**Ref-set**	**Eval-set**	**WRange**	**RMSD**	**r**	**RMSD**	**r**	**RMSD**	**r**	**RMSD**	**r**

**CDSSTR**	43	29	190–240	0.064	0.929	0.081	0.704	0.067	0.462	0.089	0.444
**CONTIN-PG**	18	18	190–240	0.05	0.96	0.06	0.94	0.1	0.31	0.11	0.49
**CONTIN-LL**	43	29	190–240	0.053	0.942	0.084	0.674	0.076	0.373	0.096	0.262
**HJ**	16	16	190–260	-	0.98	-	-0.27	-	0.18	-	0.24
**SELCON3**	43	29	190–240	0.051	0.953	0.086	0.659	0.073	0.382	0.11	0.181
**SOMCD**	45	39	190–240	0.07	0.95	0.08	0.92	0.04	0.75	0.06	0.94
**VARSEL**	16	16	190–260	-	0.95	-	0.45	-	0.54	-	0.69

Labels as in table 1, T: beta turns, U: unordered. CDSSTR, CONTIN-LL, and SELCON3 performance values are taken from [11], CONTIN-PG values from [5], HJ values from [4], SOMCD values from [12], and VARSEL values from [13]

K2D2 compares well with other published methods for prediction of protein secondary structure from CD spectra (see Table 2). We note, however, that the performance values are not readily comparable across methods because they have been trained and evaluated with different datasets (See the effect of this on K2D's performance in Table 1). Moreover, performance from methods that predict different number of secondary structure types are also not comparable because the variance of the predictions for methods that predict more types would be smaller as the predictions are normalized.

In any case, K2D2 and its predecessor, K2D, have a feature that we believe make them unique when compared to other methods, that is to warn conclusively the user when the prediction is not reliable according to the similarity between the user's input spectrum and the one computed from the training set. In summary, we believe that K2D2 represents a significant improvement and we have strived to make it easy to access and to use. We encourage users to provide suggestions for further improvements and to share novel CD spectra of proteins of known structures that can be used by us and by other developers of similar methods to improve the accuracy of the predictions.

Finally, since other methods might present alternative features not considered by us and since the benchmark results are apparently not that different, we recommend users to follow recent bibliography to see which prediction programs are used by colleagues doing similar type of analyses, and to try more than one method if the results of the predictions are unconvincing.

## Availability and requirements

Project name: K2D2

Project home page: 

Operating systems: Platform independent

Programming language: Perl

## List of abbreviations used

CD: Circular Dichroism; nm: nanometers; r: correlation coefficient; RMSD: root mean square deviation; SOM: Self Organizing Map; PDB: Protein Data Bank.

## Authors' contributions

CP–I and MAA–N participated in the development and testing of K2D2, the web server implementation and in the manuscript's preparation. Both authors read and approved the final manuscript.
